# Immunotherapy after progression to double immunotherapy: pembrolizumab and lenvatinib versus conventional chemotherapy for patients with metastatic melanoma after failure of PD-1/CTLA-4 inhibition

**DOI:** 10.3389/fonc.2024.1420879

**Published:** 2024-10-07

**Authors:** Georgios Lyrarakis, Michael Liontos, Amalia Anastasopoulou, Spyridon Bouros, Aikaterini Gkoufa, Panagiotis Diamantopoulos, Helen Gogas, Dimitrios C. Ziogas

**Affiliations:** ^1^ First Department of Medicine, National and Kapodistrian University of Athens, School of Medicine, Laiko General Hospital, Athens, Greece; ^2^ Department of Clinical Therapeutics, Alexandra General Hospital, National and Kapodistrian University of Athens, School of Medicine, Athens, Greece

**Keywords:** lenvatinib, pembrolizumab, anti-CTLA-4, anti-PD-1, double immunotherapy, melanoma, immunotherapy failure

## Abstract

**Background:**

Programmed cell death 1 receptor (PD-1) inhibition as monotherapy followed by Cytotoxic T-lymphocyte associated protein 4 (CTLA-4) inhibition in case of progression or as upfront double co-inhibition has drastically improved the survival outcomes of metastatic melanoma. Still, many patients develop primary or acquired resistance to both agents, relapse soon, and survive less. For these patients, the therapeutic options are very limited, and for many years, conventional chemotherapy (CC) was the standard of care. Recently, the phase II LEAP-004 trial supported that pembrolizumab/lenvatinib could potentially overcome anti-PD-1/anti-CTLA-4 immunotherapy refractoriness.

**Materials and methods:**

In the absence of any prospective comparative study and to evaluate in a real-world context the clinical benefit of re-administering a PD-1 inhibitor (pembrolizumab 200 mg i.v. every 3 weeks, Q3W) with a multi-kinase inhibitor (lenvatinib, but at a reduced dose 10 mg p.o. daily due to its known toxicity) in this frail population of unmet need, we conducted here a retrospective comparison of LEAP-004-proposed combination with CC (carboplatin 4 AUC and dacarbazine 850 mg/m^2^ i.v. Q3W) in melanoma patients who relapsed to both checkpoint inhibitors, either in combinatorial or in sequential setting, between July 2022 and January 2024. Baseline demographics, disease characteristics, and treatment outcomes (objective response rate (ORR), progression-free survival (PFS), and overall survival (OS)) were recorded. Survival analyses were performed using the Kaplan–Meier method. All patients were also considered for safety analysis.

**Results:**

A total of 84 patients were included in the effectiveness and safety analysis (pembrolizumab/lenvatinib, n=39 and CC, n=45). The median age was 67 (45–87) years and 64 (34–87) years, and men were 33.3% and 46.7%, respectively. The distribution of their metastatic sites was comparable, including 12.8% and 20% with brain involvement. Most patients had a good PS<2 (69.9% and 56.5%), increased lactate dehydrogenase (LDH) (71.8% and 84.4%), *BRAF*-wild status (82.1% and 84.8%), and received ≥2 previous systemic therapies (61.5% and 53.3%). The median follow-up was 18 months. The ORR was 23.1% and 11.1% (p<0.0001), the median PFS was 4.8 months and 3.8 months [HR (95%CI), 0.57 (0.36–0.92); p=0.017], and the median OS was 14.2 months and 7.8 months [HR (95%CI), 0.39 (0.22–0.69), p=0.0009] in pembrolizumab/lenvatinib and CC arms, respectively. Grade 3–5 treatment-related adverse events were documented in 48.7% (pembrolizumab/lenvatinib) and 75.6% (CC) of patients (p=0.034), which led to treatment discontinuation in 10.3% and 17.8% of cases, respectively.

**Conclusions:**

This is the first comparative study in patients with metastatic melanoma refractory to PD-1/CTLA-4 inhibition and showed significantly longer outcomes in cases treated with pembrolizumab/lenvatinib versus CC.

## Introduction

Unleashing the adaptive immunity with immune checkpoint inhibitors (ICIs) has drastically improved the clinical outcomes of patients with advanced or metastatic melanoma. More than half of melanoma patients receiving an anti-PD-1 agent as adjuvant monotherapy survive free of their disease after 5 years (5-year relapse-free survival, RFS%, for pembrolizumab, 55.4% and for nivolumab, 51.7%) (1, 2), while in the metastatic setting, pembrolizumab had a 5-year overall survival rate (OS%) of 41% in treatment-naive patients (3), while nivolumab and ipilimumab combination reached an OS% of 49% at a median follow-up of 6.5 years (4). Still, a high proportion of treated patients develop primary or acquired resistance to both agents, derive no benefit from immunotherapy, relapse soon, and survive less (5). For these cases who are resistant to anti-PD-1 monotherapy, having an activating *BRAF* mutation, targeted therapy (TT) with a BRAF-MEK combination is the best second line option (6, 7), while for those without a V600 *BRAF* mutation or developing resistance to TT, the usual next line treatment was ipilimumab [alone or in combination (8–10) or conventional chemotherapy (CC) (when Ipilimumab has already been used)]. Therefore, treatment options for melanoma patients who relapse to both anti-PD-1 and anti-CTLA4 ICIs after their combinatorial or sequential administration remain limited for many years.

Recently, the phase II LEAP-004 trial supported that the combination of pembrolizumab and lenvatinib could potentially overcome anti-PD-1/anti-CTLA-4 immunotherapy refractoriness. LEAP-004 (NCT03776136) is an open-label, single-arm, phase II study of lenvatinib [20 mg once daily (OD)] together with pembrolizumab [200 mg every 3 weeks (Q3W) for 35 doses] in patients previously treated with an anti-PD-1 ICI, either alone or in combination with ipilimumab (an anti-CTLA4 ICI) (11, 12). LEAP-004 study enrolled 103 patients with unresectable stage III or IV melanoma who progressed during treatment or within 3 months of last dose of an anti-PD-1 ICI given alone or in combination. Of the patients, 42% have received only one prior line and the rest 58% had received two or more previous lines. Only 30 of LEAP-004 patients had progressive disease after failure of PD-1/CTLA-4 inhibition with an ORR of 33.3% and median PFS (mPFS) and OS (mOS) of 4.2 months and 14 months, respectively, while at 12 months, 17.8% of cases were progression free and 54.5% were alive (11, 12).

To build on the numbers of 30 patients of LEAP-004 cohort considered refractory to nivolumab/ipilimumab, we collected here our experience using pembrolizumab/lenvatinib in these heavily pre-treated melanoma patients that relapsed to both anti-PD-1 and anti-CTLA-4 ICIs either in combinatorial or in sequential setting. In parallel, we conducted a retrospective analysis in a real-world context comparing the results of pembrolizumab/lenvatinib combination with the efficacy and toxicity of conventional chemotherapy regimen of carboplatin and dacarbazine in this difficult-to-treat melanoma population.

## Materials and methods

### Study design and patients

We retrospectively reviewed the electronic medical records of all melanoma patients treated at our site (Oncology Department, Laiko General Hospital, National and Kapodistrian University of Athens, School of Medicine, Athens, Greece) with the combination of pembrolizumab and lenvatinib between July 2022 and January 2024. For the same period, we collected also the data of melanoma patients treated at our site with CC after progression to both ICIs, either in combinatorial or in sequential setting. Included patients were adults (>18 years old), with histologically or cytologically confirmed unresectable melanoma [stage III or IV, ≥1 lesion measurable according to iRECIST v1.1 (13)] not amenable to local therapy and previously relapsed to an anti-PD-1 ICI and to an anti-CTLA-4 ICI either in combinatorial or in sequential setting. Patients with active central nervous system (CNS) disease were excluded; patients with brain metastases were eligible if they were previously untreated but asymptomatic, or previously treated with clinically stable CNS disease and no need for steroids before pembrolizumab/lenvatinib initiation. Patients with ocular melanoma were not excluded. Their melanoma should progress on immunotherapy or within 12 weeks of the last dose of anti-PD-1 therapy or anti-CTLA4 therapy given alone or in combination for at least two doses. Patients had been informed then by their physicians about the standard of care (e.g., conventional chemotherapy) and the investigational option of immunotherapy rechallenge (e.g., pembrolizumab and lenvatinib) and, after a comprehensive discussion, made a shared treatment decision. Pembrolizumab was administered at 200 mg intravenously every 3 weeks (Q3W), but lenvatinib was started at a reduced dose of 10 mg orally once daily (OD) due to its known toxicity (grade ≥3 AEs in 45.6% of patients included in LEAP-004 study). In cases experiencing severe toxicity related to lenvatinib, the recommended dose reductions were 8 mg OD (at the first reduction) and 4 mg OD (at the second reduction). Conventional chemotherapy included carboplatin 4 AUC and dacarbazine 850 mg/m^2^ i.v. Q3W and dose reduction followed the dosing guidelines. Baseline demographics, melanoma-related characteristics, and treatment outcomes (ORR, PFS, and OS) and side effects were recorded. Treatment was continued until melanoma progression, unacceptable toxicity, or after investigator decision. Patients who experienced intolerable toxicity attributed to one drug discontinued that drug and continued the other.

### Assessments and statistical analysis

*BRAF* V600 mutation status was assessed for all patients during screening. Tumor imaging was scheduled at baseline and every 12 weeks including intracranial response assessment using brain magnetic resonance imaging on the same schedule. Radiological response was described per immune response evaluation criteria, iRECIST v1.1 (13). Clinical assessment for treatment-related adverse events (TRAEs) and laboratory abnormalities was recorded during and up to 90 days after treatment discontinuation and graded according to the National Cancer Institute Common Terminology Criteria for Adverse Events (CTCAE) version 5 (14, 15). The entire cohort was fairly homogeneous and relatively small, and no matching was performed. The major patients’ baseline parameters were compared with unmatched analyses (e.g., Mann–Whitney U test and Fisher’s exact test). Efficacy and safety were assessed in all included patients. The survival times, PFS and OS, were estimated using the Kaplan–Meier method, and the 95% exact binomial CIs for ORR were calculated using the Clopper–Pearson method. Data were handled in accordance with the local IRB approval. All statistical analyses were done with GraphPad version 5.

## Results

A total of 84 patients who received at least one cycle of pembrolizumab/lenvatinib or CC were included in the final analysis (pembrolizumab/lenvatinib, n=39, and CC, n=45). All patients had access to lenvatinib via request to the national health system, outside of a clinical trial setting. Baseline demographics and melanoma-related parameters are presented in **Table 1**. The median age was 67 (45–87) years and 64 (34–87) years, and men were 33.3% and 46.7%, respectively. Patients with different melanoma subtypes were represented in both treatment groups: cutaneous (72% vs. 75%), acral (13% vs. 14%), mucosal (8% vs. 9%), uveal (5% vs. 2%), and unknown primary melanoma (3% vs. 0%). Seven patients in each group had a *BRAF* V600 mutation (7/39, 17.9% and 7/45, 15.2%, respectively), and the other 32 and 38 patients had wild-type (WT) *BRAF* (82.1% and 84.8%, respectively). Their melanoma staging was comparable, and at treatment initiation, 12.8% and 20% of patients had brain involvement. Most patients had a good Eastern Cooperative Oncology Group (ECOG) performance status. (PS<2) (69.9% and 56.5%). Baseline levels of lactate dehydrogenase (LDH) were increased for 71.8% and 84.4% (35.9% and 33.3% elevated more than twice the upper normal limit, >2XUNL). Similar proportions of patients received ≥2 previous systemic therapies (61.5% and 53.3%). In the pembrolizumab/lenvatinib group, 56.4% had been concurrently exposed in nivolumab/Ipilimumab combination and 43.6% had relapsed after treatment with anti-PD-1 and anti-CTLA4 ICI in subsequent lines. Similarly, in the CC group, 55.6% had been concurrently exposed in nivolumab/Ipilimumab combination and 44.4% in anti-PD-1 and anti-CTLA4 treatments in sequential setting. The median follow-up was 18 months.

**Table 1:** Baseline Demographics and Disease Characteristics of Pembrolizumab/Lenvatinib and conventional chemotherapy arms.

	Pembrolizumab+Lenvatinib Group (n=39)	Standard chemotherapy Group (n=45)	P-value (Mann-Whitney, two-tailed)
Males, number (%)	13 (33.3)	21 (46.7)	0.267
Age, median, years (range)	67 (40–87)	64 (34–87)	0.892
ECOG PS >=2, number (%)	12 (30.1)	20 (43.5)	0.261
Stage IV, M1a, number (%)	10 (25.6)	12 (26.7)	>0.999
Stage IV, M1b, number (%)	8 (20.5)	10 (22.2)	>0.999
Stage IV, M1c, number (%)	16 (41.0)	14 (31.1)	0.370
Stage IV, M1d, number (%)	5 (12.8)	9 (20.0)	0.559
BRAF mutated status, number (%)	7 (17.9)	7 (15.2)	>0.999
LDH >ULN, number (%)	28 (71.8)	38 (84.4)	0.189
Previous therapies >=2, number (%)	24 (61.5)	24 (53.3)	0.511
Nivolumab/Ipilimumab combination, number (%)	22 (56.4)	25 (55.6)	>0.999
Anti-PD-1+anti-CTLA-4 in sequent lines, number (%)	17 (43.6)	20 (44.4)	>0.999

**Figure 1:**
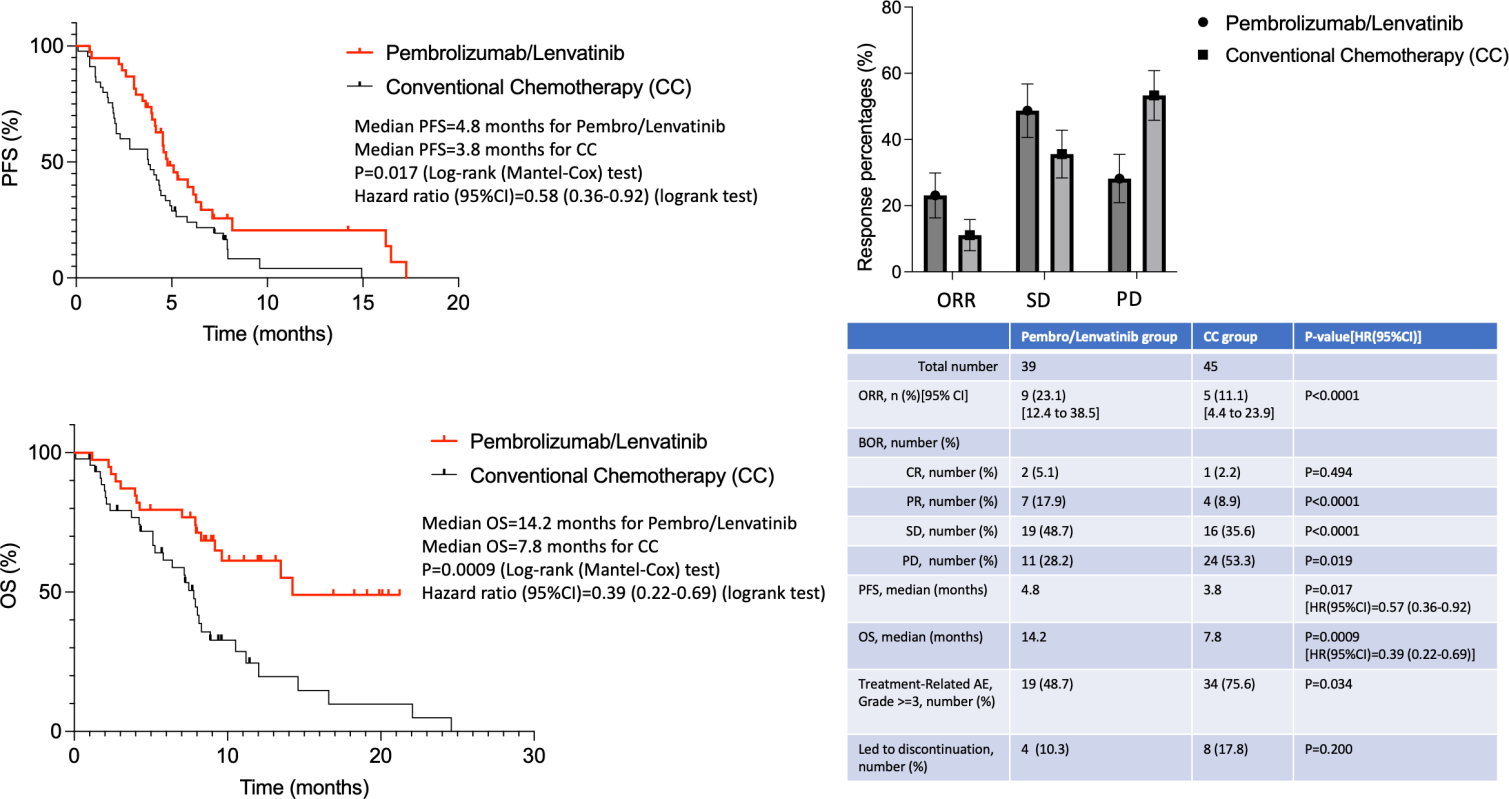
Efficacy and toxicity outcomes of pembrolizumab/lenvatinib and standard chemotherapy arms.

Response assessment was done using imaging for all patients, irrespective of their clinical improvements or deteriorations. For cases with CNS melanoma disease, the response was evaluated both systemically and intracranially (using brain imaging at baseline and at every response assessment). The best ORR was 23.1% in the pembrolizumab/lenvatinib group and 11.1% in the CC group (p<0.0001), respectively (**Figure 1**). More specifically, 18.0% achieved PR and 5.1% CR and another 38.5% achieved SD, leading to a total of 61.6% disease control rate (DCR) in the pembrolizumab/lenvatinib group, while 8.9% achieved PR and 2.2% CR and another 35.6% achieved SD, leading to a total of 46.7% DCR. The mOS for pembrolizumab/lenvatinib and CC arms was 14.2 months and 7.8 months [HR (95%CI): 0.39 (0.22–0.69); p<0.009], and the mPFS was 4.8 months and 3.8 months [HR (95%CI): 0.57 (0.36–0.92); p=0.017], respectively (**Figure 1**). The median duration of response was 4.5 months (range, 2–21), with two patients still ongoing at data cutoff. All patients with CNS melanoma involvement were simultaneously treated with stereotactic radiosurgery (SRS) before initiating systemic therapy with pembrolizumab/lenvatinib or CC, and none of them underwent a neurosurgical intervention. The intracranial response rate reached to 17% and 9% in pembrolizumab/lenvatinib and CC groups, respectively. In both arms, more than half had also intracranial disease progression at the time of systemic PD. Responses according to melanoma subtypes have not been compared, as the numbers are too small and no strong conclusions for these rarer melanoma sub-cohorts are possible. For instance, one of the two uveal melanoma patients in the pembrolizumab/lenvatinib group had stable disease at first imaging assessment and progressive disease at the second imaging assessment, while the other case had progression of uveal melanoma at first imaging assessment and no response observed in the individual with uveal melanoma in the CC arm. Outcomes and responses are described in **Figure 1**.

As already known, the combination of pembrolizumab/lenvatinib was quite toxic with 48.7% of patients developing grade 3–4 treatment-related adverse events (TRAEs), but still lower compared to the 75.6% CC-treated patients (p=0.034) (**Figure 1**). The most common grade 3–4 TRAEs were hypertension and diarrhea. Only two patients developed grade 3 immune-mediated hypothyroidism, and no patients had grade 4 or 5 potentially immune-mediated AEs. A total of 67 (67.3%) patients had ≥1 lenvatinib dose reduction, while 10.3% and 17.8% of cases had to stop their treatment due to toxicity in the two treatment arms, respectively. Three (7.7%) patients died from pembrolizumab/lenvatinib-related AEs (one from decreased platelet count, one from VTE, and one from sepsis), and seven (15.5%) patients died from toxicity in the CC arm (five from neutropenia/sepsis and two from VTE).

## Discussion

This study validates the antitumor activity of the combination of pembrolizumab and lenvatinib in real-world patients with metastatic melanoma with confirmed progression on previous anti-PD-1/CTLA-4 therapy and shows for first time the superiority of this combination over chemotherapy. Our results for pembrolizumab/lenvatinib combination are comparable with respect to previous ones of phase II, single-arm, open-label, LEAP-004 trial. The LEAP-004 trial, even from its initial announcement at virtual ESMO 2020, showed that the combination of pembrolizumab/lenvatinib had a promising ORR of 21.4% (31% in patients that had progressed to prior anti-PD-1/anti-CTLA-4 combination). Longer follow-up data presented at ASCO 2021 (median follow-up of 15.3 months) reconfirmed the therapeutical benefit: ORR remained stable over time (21.4%) and comparable with previous analysis (11, 12). Interestingly, ORR had slightly increased from 31% to 33.3% in the subgroup of 30 patients with PD on prior anti-PD-1/anti-CTLA-4 immunotherapy while for the entire study population, mPFS and mOS remained unchanged at 4.2 months and 14 months, respectively, with 17.8% of patients being progression-free, and 54.5% being alive at 12 months (11, 12). The ORR of pembrolizumab/lenvatinib in our study was 23.1%, slightly worse than LEAP-004 anti-PD-1/anti-CTLA-4 refractory subgroup; probably due to the nature of our real-world, unselected and unfit for a clinical trial, population and the reduced initiating dose of lenvatinib, even though the mPFS (4.8 months) and the mOS (14.2 months) were similar to LEAP-004 survival times. In another paper recording the real-world evidence of pembrolizumab/lenvatinib administration (with lenvatinib at 20 mg/day) as an advanced line for metastatic melanoma, Stoff et al. reported responses across lines of treatment, with a 25% ORR in the second and third lines and a 36% ORR in the fourth and fifth lines, with a mPFS of 3 months and a mOS of 11 months (16).

Regarding safety, pembrolizumab/lenvatinib combination confirms its high toxicity rates, even at a reduced dose of lenvatinib (10 mg/day). Grade ≥3 TRAEs, most commonly hypertension and diarrhea, were documented in 48.7% of patients treated with pembrolizumab/lenvatinib, leading to treatment discontinuation in 10.3% of cases and to death in 7.7%, respectively. These percentages are higher compared to those reported in LEAP-004 (11, 12), and it might be also attributed to the frailty of our study population, while Stoff et al. reported a lower grade ≥3 toxicity rate, but its study included not older than 80 years old patients with smaller proportions of CNS involvement and elevated LDH (16). In the CC arm, our safety findings were similarly high with an old study by Agarwala et al., where 28 patients who received carboplatin 300 mg/m^2^ and dacarbazine 1,000 mg/m^2^ i.v. Q3W experienced 21 events of hematologic grade ≥3 toxicities and two events of non-hematologic grade ≥3 toxicities (23/28, 82.1%) (17).

A deeper understanding of the underlying biology of ICI-resistance phenotypes would enable better selection of patient subgroups and immunotherapy decisions (18). Pre-treatment analyses of possible molecular biomarkers for immunotherapy sensitivity or resistance are still needed (Ziogas et Pali, *Frontiers in Immunology* 2024). For instance, high levels of an angiogenesis gene signature score at baseline, low baseline serum angiopoietin-2, or early induction of hypertension during treatment could identify responders and could help in a better selection of patients for future anti-VEGFR therapies (19). In metastatic melanoma, anti-angiogenesis drugs combined with chemotherapy (e.g., bevacizumab/dacarbazine combination) have been used and demonstrated similar efficacy results, although in a different setting of untreated patients: ORR was 18.9% and clinical benefit was 48.6%. mPFS was 5.5 months with a mOS of 11.4 months (20). Lenvatinib targets several cancer-associated pathways, including the VEGFR and the fibroblast growth factor receptor (FGFR). Based on pre-clinical evidence, mainly in mouse models, this blockade shifts the tumor microenvironment to an immune-stimulatory status offering to the combination greater antitumor activity than either agent alone (21–25). Lenvatinib has shown a low ORR of 9% when given as a single agent for previously treated metastatic melanoma patients (26), and its use as a single agent in melanoma was not pursued. Following the rationale of pembrolizumab/lenvatinib combination, a phase Ib/II open-label study 111/KEYNOTE-146 included various types of malignancies and patient numbers in its cohorts. At week 24, ORR of combination was 63% for renal cell carcinoma (19 of 30 patients), 52% for endometrial cancer (12 of 23 patients), 48% for melanoma (10 of 21 patients), 36% for head and neck squamous cell carcinoma (8 of 22 patients), 33% for non-small-cell lung cancer (7 of 21 patients), and 25% for urothelial cancer (5 of 20 patients). In this study, the maximum tolerated dose for lenvatinib was estimated at 20 mg/day with pembrolizumab at 200 mg i.v. once Q3W (27). The most common TRAEs across tumor types were fatigue (58%), diarrhea (52%), and hypertension (47%). Based on the results of KEYNOTE-146 study, the combination of pembrolizumab/lenvatinib received accelerated approvals in some countries for previously treated endometrial carcinoma (irrespectively of microsatellite instability or mismatch repair status) (28, 29) and entered into the focus of numerous trials across different cancer types (e.g., ovarian cancer, renal cancer, and hepatocarcinoma) with encouraging preliminary results (22, 25, 27, 29–32).

## Conclusions

Our single institution experience, recorded here, shows for first time the superiority of pembrolizumab/lenvatinib combination over the CC option in melanoma patients after progression to double immunotherapy and gives some encouraging insights for this population with unmet medical needs. Yet, its results should be interpreted with caution and could not be generalized across specific melanoma subgroups. To comprehensively address questions regarding rarer melanoma subtypes or to recognize influencing factors in previous lines of treatment that could affect the outcomes of pembromizumab/lenvatinib, it is recommended to expand the sample size and increase the number of participating research sites. Undeniably, the presented study is a retrospective analysis of a single institutionand not a prospective multicenter randomized controlled trial in a small difficult-to-treat subpopulation. Our study cohorts include heavily pre-treated patients (e.g., received ≥2 previous systemic therapies, pre-exposed to both anti-PD-1 and anti-CTLA4 ICIs), with an aggressive melanoma disease (e.g., characterized by elevated LDH and *BRAF* mutated status) and high rates of CNS involvement (e.g., M1c disease). The diversity and heterogeneity of this population gives a more comprehensive real-world picture of multi-treated melanoma patients than their depiction in clinical trials, supporting this regimen’s potential over chemotherapeutic options in later treatment lines for metastatic melanoma. More data are coming over different cancer types for the utility of this regimen in ICI-refractory malignancies, while the mature results of LEAP-004 study and a randomized phase III trial with an appropriate comparator arm may help the allocation of pembrolizumab/lenvatinib combination in the algorithm of metastatic melanoma treatment.

## Data Availability

The original contributions presented in the study are included in the article/supplementary material. Further inquiries can be directed to the corresponding author.

## References

[1] Ascierto PA Del Vecchio M Mandala M Gogas H Arance AM Dalle S. Adjuvant nivolumab versus ipilimumab in resected stage iiib-C and stage iv melanoma (Checkmate 238): 4-year results from a multicentre, double-blind, randomised, controlled, phase 3 trial. Lancet Oncol. (2020) 21:1465–77. doi: 10.1016/S1470-2045(20)30494-0 32961119

[2] Eggermont AMM Blank CU Mandala M Long GV Atkinson VG Dalle S. Adjuvant pembrolizumab versus placebo in resected stage iii melanoma (Eortc 1325-mg/keynote-054): distant metastasis-free survival results from a double-blind, randomised, controlled, phase 3 trial. Lancet Oncol. (2021) 22:643–54. doi: 10.1016/S1470-2045(21)00065-6 33857412

[3] Hamid O Robert C Daud A Hodi FS Hwu WJ Kefford R. Five-year survival outcomes for patients with advanced melanoma treated with pembrolizumab in keynote-001. Ann Oncol. (2019) 30:582–8. doi: 10.1093/annonc/mdz011 PMC6503622 30715153

[4] Wolchok JD Chiarion-Sileni V Gonzalez R Grob JJ Rutkowski P Lao CD. Long-term outcomes with nivolumab plus ipilimumab or nivolumab alone versus ipilimumab in patients with advanced melanoma. J Clin Oncol. (2022) 40:127–37. doi: 10.1200/JCO.21.02229 PMC8718224 34818112

[5] Ziogas DC Theocharopoulos C Koutouratsas T Haanen J Gogas H. Mechanisms of resistance to immune checkpoint inhibitors in melanoma: what we have to overcome? Cancer Treat Rev. (2023) 113:102499. doi: 10.1016/j.ctrv.2022.102499 36542945

[6] Atkins MB Lee SJ Chmielowski B Tarhini AA Cohen GI Truong TG. Combination dabrafenib and trametinib versus combination nivolumab and ipilimumab for patients with advanced braf-mutant melanoma: the dreamseq trial-ecog-acrin ea6134. J Clin Oncol. (2023) 41:186–97. doi: 10.1200/JCO.22.01763 PMC9839305 36166727

[7] Ascierto PA Mandala M Ferrucci PF Guidoboni M Rutkowski P Ferraresi V. Sequencing of ipilimumab plus nivolumab and encorafenib plus binimetinib for untreated braf-mutated metastatic melanoma (Secombit): A randomized, three-arm, open-label phase ii trial. J Clin Oncol. (2023) 41:212–21. doi: 10.1200/JCO.21.02961 36049147

[8] Friedman CF Spencer C Cabanski CR Panageas KS Wells DK Ribas A. Ipilimumab alone or in combination with nivolumab in patients with advanced melanoma who have progressed or relapsed on pd-1 blockade: clinical outcomes and translational biomarker analyses. J Immunother Cancer. (2022) 10(1):e003853. doi: 10.1136/jitc-2021-003853 35074903 PMC8788323

[9] Bowyer S Prithviraj P Lorigan P Larkin J McArthur G Atkinson V. Efficacy and toxicity of treatment with the anti-ctla-4 antibody ipilimumab in patients with metastatic melanoma after prior anti-pd-1 therapy. Br J Cancer. (2016) 114:1084–9. doi: 10.1038/bjc.2016.107 PMC4865968 27124339

[10] Zimmer L Apuri S Eroglu Z KottsChade LA Forschner A Gutzmer R. Ipilimumab alone or in combination with nivolumab after progression on anti-pd-1 therapy in advanced melanoma. Eur J Cancer. (2017) 75:47–55. doi: 10.1016/j.ejca.2017.01.009 28214657

[11] Arance A de la Cruz-Merino L Petrella TM Jamal R Ny L Carneiro A. Phase ii leap-004 study of lenvatinib plus pembrolizumab for melanoma with confirmed progression on a programmed cell death protein-1 or programmed death ligand 1 inhibitor given as monotherapy or in combination. J Clin Oncol. (2023) 41(1):75–85. doi: 10.1200/JCO.22.00221 35867951

[12] Arance A de la Cruz-Merino L Petrella TM Jamal R Ny L Carneiro A. Phase ii leap-004 study of lenvatinib plus pembrolizumab for melanoma with confirmed progression on a programmed cell death protein-1 or programmed death ligand 1 inhibitor given as monotherapy or in combination. J Clin Oncol. (2023) 41:75–85. doi: 10.1200/JCO.22.00221 35867951

[13] Seymour L Bogaerts J Perrone A Ford R Schwartz LH Mandrekar S. Irecist: guidelines for response criteria for use in trials testing immunotherapeutics. Lancet Oncol. (2017) 18:e143–e52. doi: 10.1016/S1470-2045(17)30074-8 PMC5648544 28271869

[14] Freites-Martinez A Santana N Arias-Santiago S Viera A. Using the common terminology criteria for adverse events (Ctcae - version 5.0) to evaluate the severity of adverse events of anticancer therapies. Actas Dermosifiliogr (Engl Ed). (2021) 112:90–2. doi: 10.1016/j.ad.2019.05.009 32891586

[15] Kohlmann J Kirsten H Simon JC Ziemer M. Refined common terminology criteria for adverse events criteria - respective systemic melanoma therapy. Melanoma Res. (2019) 29:444–5. doi: 10.1097/CMR.0000000000000554 31246725

[16] Stoff R Asher N Laks S Steinberg Y Schachter J Shapira-Frommer R. Real world evidence of lenvatinib + Anti pd-1 as an advanced line for metastatic melanoma. Front Oncol. (2023) 13:1180988. doi: 10.3389/fonc.2023.1180988 37274272 PMC10233023

[17] Agarwala SS Ferri W Gooding W Kirkwood JM. A phase iii randomized trial of dacarbazine and carboplatin with and without tamoxifen in the treatment of patients with metastatic melanoma. Cancer. (1999) 85:1979–84. doi: 10.1002/(sici)1097-0142(19990501)85:9<1979::aid-cncr15>3.0.co;2-g 10223239

[18] Ziogas DC Theocharopoulos C Koutouratsas T Haanen J Gogas H. Mechanisms of resistance to immune checkpoint inhibitors in melanoma: what we have to overcome? Cancer Treat Rev. (2022) 113:102499. doi: 10.1016/j.ctrv.22022.102499 36542945

[19] Ferrucci PF. Lenvatinib/pembrolizumab as second line treatment for advanced melanoma patients refractory to programmed death 1 (Pd-1)/programmed death ligand-1 (Pd-L1) inhibitors. Ann Transl Med. (2023) 11:296. doi: 10.21037/atm-23-341 37181342 PMC10170280

[20] Ferrucci PF Minchella I Mosconi M Gandini S Verrecchia F Cocorocchio E. Dacarbazine in combination with bevacizumab for the treatment of unresectable/metastatic melanoma: A phase ii study. Melanoma Res. (2015) 25:239–45. doi: 10.1097/CMR.0000000000000146 25746039

[21] Kimura T Kato Y Ozawa Y Kodama K Ito J Ichikawa K. Immunomodulatory activity of lenvatinib contributes to antitumor activity in the hepa1-6 hepatocellular carcinoma model. Cancer Sci. (2018) 109:3993–4002. doi: 10.1111/cas.13806 30447042 PMC6272102

[22] Kato Y Tabata K Kimura T Yachie-Kinoshita A Ozawa Y Yamada K. Lenvatinib plus anti-pd-1 antibody combination treatment activates cd8+ T cells through reduction of tumor-associated macrophage and activation of the interferon pathway. PloS One. (2019) 14:e0212513. doi: 10.1371/journal.pone.0212513 30811474 PMC6392299

[23] Deng H Kan A Lyu N Mu L Han Y Liu L. Dual vascular endothelial growth factor receptor and fibroblast growth factor receptor inhibition elicits antitumor immunity and enhances programmed cell death-1 checkpoint blockade in hepatocellular carcinoma. Liver Cancer. (2020) 9:338–57. doi: 10.1159/000505695 PMC7325120 32647635

[24] Adachi Y Kamiyama H Ichikawa K Fukushima S Ozawa Y Yamaguchi S. Inhibition of fgfr reactivates ifngamma signaling in tumor cells to enhance the combined antitumor activity of lenvatinib with anti-pd-1 antibodies. Cancer Res. (2022) 82:292–306. doi: 10.1158/0008-5472.CAN-20-2426 34753772 PMC9397636

[25] Yi C Chen L Lin Z Liu L Shao W Zhang R. Lenvatinib targets fgf receptor 4 to enhance antitumor immune response of anti-programmed cell death-1 in hcc. Hepatology. (2021) 74:2544–60. doi: 10.1002/hep.31921 34036623

[26] O’Day S Gonzalez R Kim K Chmielowski B Kefford R Long G. A phase ii study of the multitargeted kinase inhibitor lenvatinib in patients with advanced braf wild-type melanoma. J Clin Oncol. (2013) 31:9026–. doi: 10.1200/jco.2013.31.15_suppl.9026

[27] Taylor MH Lee CH Makker V Rasco D Dutcus CE Wu J. Phase ib/ii trial of lenvatinib plus pembrolizumab in patients with advanced renal cell carcinoma, endometrial cancer, and other selected advanced solid tumors. J Clin Oncol. (2020) 38:1154–63. doi: 10.1200/JCO.19.01598 PMC7145588 31961766

[28] Arora S Balasubramaniam S Zhang W Zhang L Sridhara R Spillman D. Fda approval summary: pembrolizumab plus lenvatinib for endometrial carcinoma, a collaborative international review under project orbis. Clin Cancer Res. (2020) 26:5062–7. doi: 10.1158/1078-0432.CCR-19-3979 32295834

[29] Makker V Taylor MH Aghajanian C Oaknin A Mier J Cohn AL. Lenvatinib plus pembrolizumab in patients with advanced endometrial cancer. J Clin Oncol. (2020) 38:2981–92. doi: 10.1200/JCO.19.02627 PMC7479759 32167863

[30] Finn RS Ikeda M Zhu AX Sung MW Baron AD Kudo M. Phase ib study of lenvatinib plus pembrolizumab in patients with unresectable hepatocellular carcinoma. J Clin Oncol. (2020) 38:2960–70. doi: 10.1200/JCO.20.00808 PMC7479760 32716739

[31] Makker V Taylor MH Aghajanian C Cohn AL Brose MS Simone CD. Evaluation of potential biomarkers for lenvatinib plus pembrolizumab among patients with advanced endometrial cancer: results from study 111/keynote-146. J Immunother Cancer. (2024) 12(1):e007929. doi: 10.1136/jitc-2023-007929 38242717 PMC10806562

[32] Motzer R Alekseev B Rha SY Porta C Eto M Powles T. Lenvatinib plus pembrolizumab or everolimus for advanced renal cell carcinoma. N Engl J Med. (2021) 384:1289–300. doi: 10.1056/NEJMoa2035716 33616314

